# 不同剂量紫杉醇联合卡铂一线治疗晚期非小细胞肺癌的临床研究

**DOI:** 10.3779/j.issn.1009-3419.2011.04.04

**Published:** 2011-04-20

**Authors:** 嘉琳 钱, 洁 沈, 皓 白, 宝惠 韩

**Affiliations:** 200030 上海，上海交通大学附属胸科医院肺内科 Department of Pulmonary Medicine, Shanghai Chest hospital, Shanghai Jiaotong University, Shanghai 200030, China

**Keywords:** 肺肿瘤, 化疗, 紫杉醇, 卡铂, Lung neoplasms, Drug therapy, Paclitaxel, Carboplatin

## Abstract

**背景与目的:**

紫杉醇联合卡铂一线治疗晚期非小细胞肺癌（non-small cell lung cancer, NSCLC）临床应用较为广泛。本研究旨在比较不同剂量紫杉醇联合卡铂一线治疗晚期NSCLC患者的毒副反应及疗效。

**方法:**

2006年12月-2008年6月，共63例晚期NSCLC患者接受紫杉醇175 mg/m^2^或200 mg/m^2^联合卡铂（AUC 5）化疗，前者42例，后者21例，ECOG评分0-1分，3周-4周重复，比较两组近期和远期疗效及毒副反应。

**结果:**

紫杉醇175 mg/m^2^与200 mg/m^2^化疗组客观有效率分别为28.57%与33.33%（*P*=0.698），中位TP为6.7个月与7个月（*P*=0.561），MST为18.7个月与19个月（*P*=0.255），1年生存率为61.9%与66.7%（*P*=0.711），2年生存率为31%与33.3%（*P*=0.852）。紫杉醇200 mg/m^2^组3/4级中性粒细胞下降发生率明显高于175 mg/m^2^组，分别为61.9%与33.3%（*P*=0.031）。

**结论:**

与200 mg/m^2^化疗组相比，紫杉醇175 mg/m^2^联合卡铂一线治疗晚期NSCLC患者可明显减少3/4级中性粒细胞下降发生率，且疗效及生存期并不劣于较高剂量化疗组。

## Abstract (en)

**Background and objective:**

The combination of pacilitaxel and carboplatin has become a widely used regimen in frst-line therapy of advanced NSCLC. The aim of this study is to compare the efcacy and safety of paclitaxel plus carboplatin at two dose levels in the frst-line treatment of advanced NSCLC patients.

**Methods:**

From Dec 2006 to Jun 2008, 63 patients (ECOG 0-1) with advanced NSCLC were administered with paclitaxel 175 mg/m^2^ (42 patients) plus carboplatin (AUC 5) or paclitaxel 200 mg/m^2^ (21 patients) plus carboplatin (AUC 5), 3-4 weeks/cycle.

**Results:**

The objective response rate of paclitaxel 175 mg/m^2^ group and paclitaxel 200 mg/m^2^ group was 28.57% *vs* 33.33% (*P*=0.698); median time to progression were 6.7 months *vs* 7.0 months (*P*=0.561); median overall survival time were 18.7 months *vs* 19.0 months (*P*=0.255); one and two-year survival rate were 61.9% *vs* 66.7% (*P*=0.711) and 31.0% *vs* 33.3% (*P*=0.852), respectively. Neutropenia 3/4 grade was 61.9% *vs* 33.3% in paclitaxel 200 mg/m^2^ group and paclitaxel 175 mg/m^2^ group (*P*=0.031).

**Conclusion:**

175 mg/m^2^ dose paclitaxel causes less neutropenia than higher dose paclitaxel. Response rate, survival rate, time to progression and survival time are not statistically signifcant in two groups.

近年随着肺癌治疗领域基础及临床研究的不断深入，抗肿瘤药物治疗的种族差异性逐渐引起临床医师的重视。目前多数肺癌治疗的临床循证医学证据来自于欧美国家对相关人群的研究，而有研究提示特定人群的肺癌患者对一些抗肿瘤药物的疗效及毒副反应存在特异性。因此，不同地区临床医师在选择治疗药物及药物剂量时可能采用不同的方案。紫杉醇联合卡铂化疗是目前治疗晚期非小细胞肺癌（non-small cell lung cancer, NSCLC）的标准一线治疗方案，临床应用较为广泛。国外紫杉醇治疗晚期NSCLC的标准剂量为200 mg/m^2^-225 mg/m^2^，出于对患者体质等因素的考虑，国内临床医师多选择偏低于上述剂量的方案进行化疗。用药剂量的差异是否会影响中国患者的生存获益及化疗相关毒副反应，何种剂量化疗更具有优势，目前尚缺乏循证医学依据。因此，我们总结分析了上海市胸科医院2006年12月-2008年6月接受175 mg/m^2^或200 mg/m^2^紫杉醇联合卡铂化疗的63例晚期NSCLC患者的临床资料，比较两种剂量紫杉醇化疗患者的疗效、疾病进展时间（time to progression, TTP）、中位生存期（median survival time, MST）、生存率及毒副反应，以评价国内患者对不同剂量紫杉醇联合卡铂化疗的耐受性与疗效。

## 1 资料与方法

### 1.1 一般资料

63例患者均为18岁以上、组织学或细胞学确诊的Ⅲb期或Ⅳ期初治NSCLC患者，ECOG（Eastern Cooperative Oncology Group）评分0分-1分，治疗前1周内均进行体格检查、血尿常规、肝肾功能、心电图等检查，确认无化疗禁忌。治疗前2周内均行胸部CT、MRI、ECT、B超等检查，确认有可测量的客观指标用于疗效评价，既往未曾接受放、化疗，排除脑转移。所有患者化疗前均签署知情同意书。紫杉醇175 mg/m^2^及200 mg/m^2^化疗组患者比例为2:1，63例研究病例具体情况见表 1。

**1:** 紫杉醇175 mg/m^2^及200 mg/m^2^联合卡铂一线治疗晚期NSCLC患者的临床特征 Comparison of patients with NSCLC characteristics between paclitaxel (175 mg/m^2^) and paclitaxel (200 mg/m^2^) plus carboplatin groups

Characteristic	Paclitaxel 175 mg/m^2^ group (*n*=42)	Paclitaxel 200 mg/m^2^ group (*n*=21)	*P*
Gender			0.571
Male	29 (69.0%)	13 (61.9%)	
Female	13 (31.0%)	8 (38.1%)	
ECOG PS			0.869
0	3（7.1%）	2 (9.5%)	
1	39（92.9%）	19 (90.5%)	
Histology			0.912
Adenocarcinoma	33 (78.6%)	17 (81.0%)	
Squamous cell carcinoma	7 (16.7%)	4 (19.0%)	
Adenocarcinoma+Squamous cell carcinoma	2 (4.7%)	0	
Stage			0.592
Ⅲb	8（19.0%）	6（28.6%）	
Ⅳ	34（81.0%）	15（71.4%）	
Age	59.6（40-74）	56.0（35-73）	0.159
Second-line chemotherapy	23 (54.8%)	12 (57.1%)	0.858
Vinorelbine/gemcitabine plus cisplatin	5 (11.9%)	3 (14.3%)	
Docetaxel	17 (40.5%)	9 (42.9%)	
Pemetrexed	1 (2.4%)	0	
Second-line or post second-line EGFR-TKI	24 (57.1%)	11 (52.4%)	0.72
Smoke	21 (50%)	10 (47.6%)	0.859
ECOG: Eastern Cooperative Oncology Group; EGFR-TKI: epidermal growth factor receptor-tyrosine kinase inhibitors.			

### 1.2 治疗方法

使用紫杉醇及卡铂（百时美施贵宝公司）联合方案化疗。紫杉醇用药前使用地塞米松、非那根预处理，紫杉醇于化疗周期第1天静脉滴注3 h，随后予卡铂（AUC 5）（AUC, area under the concentration time curve，血药浓度-时间曲线下面积）静脉滴注30 min-60 min。每21天至28天为1个周期，计划化疗4个-6个周期。两组均不使用预防性升白细胞治疗。治疗期间根据需要使用粒细胞集落刺激因子、白介素11、止吐、抗感染等对症支持治疗。

### 1.3 疗效及毒性反应评定标准

使用RECIST（Response Evaluation Criteria in Solid Tumors）标准^[1]^评价疗效：完全缓解（complete response, CR）、部分缓解（partial response, PR）、稳定（stable disease, SD）和进展（progression of disease, PD），客观有效率（objective response rate, ORR=CR+PR）。按NCI常见毒性分级标准（CTC 3.0版）评价毒副反应。每周复查2次血常规，每周期复查肝肾功能、心电图、B超及CT，每2个化疗周期评价疗效直至疾病进展。

### 1.4 统计分析

采用SPSS 11.0软件进行统计分析，卡方检验、*Fisher’s*精确检验及秩和检验比较组间临床特征、疗效及毒副反应的差异；应用*Kaplan-Meier*单因素方法进行生存分析，*Log-rank*检验判断生存情况差异。*P* < 0.05为差异具有统计学意义。

## 2 结果

随访至2010年6月，无失访，随访截止时紫杉醇175 mg/m^2^化疗组有6人存活，200 mg/m^2^化疗组有2人存活。175 mg/m^2^化疗组平均化疗次数为4.3周期，200 mg/m^2^化疗组平均化疗次数为4.1周期。

### 2.1 疗效

紫杉醇175 mg/m^2^化疗组获PR 12例、SD 21例、PD 9例，ORR为28.57%；200 mg/m^2^化疗组获PR 7例、SD 9例、PD 5例，ORR为33.33%，两组比较无统计学差异（*P*=0.698）。见表 2。

**2:** 醇175 mg/m^2^及200 mg/m^2^组患者临床疗效、疾病进展时间及生存期比较 Comparison of response rate, median time to progression, median survival time and survival rates between paclitaxel (175 mg/m^2^) and paclitaxel (200 mg/m^2^) groups

Item	Paclitaxel 175 mg/m^2^ group (*n* =42)	Paclitaxel 200 mg/m^2^ group (*n* =21)	*P*
Response rate Complete Response (*n*)	0	0	
Partial Response (*n*)	12 (28.57%)	7 (33.33%)	
Stable disease (*n*)	21 (50.00%)	9 (42.86%)	
Progression of disease (*n*)	9 (21.43%)	5 (23.81%)	
Objective response rate (%)	12 (28.57%)	7 (33.33%)	0.698
Median time to progression (month)	6.7	7.0	0.561
Median survival time (month)	18.7	19.0	0.255
1-year survival rate (%)	61.9%	66.7%	0.711
2-year survival rate (%)	31.0%	33.3%	0.852

### 2.2 生存情况

紫杉醇175 mg/m^2^化疗组中位TTP为6.7个月，200 mg/m^2^化疗组中位TTP为7个月，两组比较无统计学差异（*P*=0.561）；175 mg/m^2^化疗组MST为18.7个月，200 mg/m^2^化疗组为19个月，两组比较无统计学差异（*P*=0.255）。1年生存率、2年生存率两组分别为61.9%、31%和66.7%、33.3%，两组比较均无统计学差异（表 2，图 1，图 2）。

**1:**
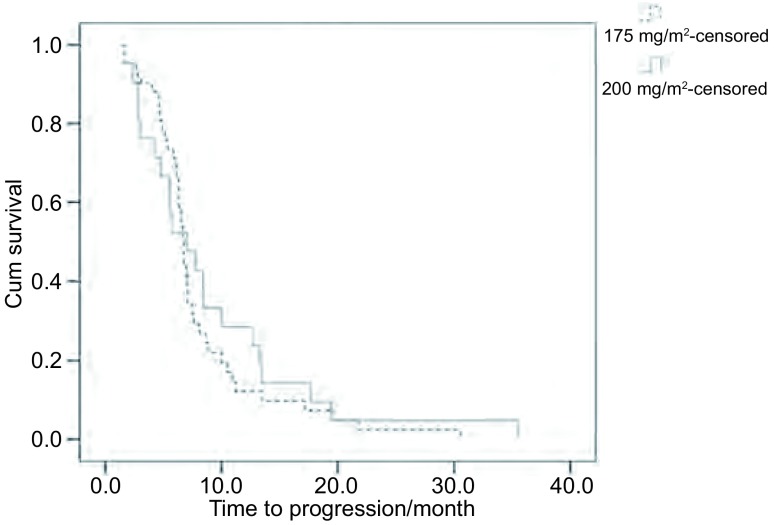
紫杉醇175 mg/m^2^及200 mg/m^2^联合卡铂化疗组患者疾病进展时间曲线 Time to progression curves of paclitaxel (175 mg/m^2^) and pacli-taxel (200 mg/m^2^) groups

**2:**
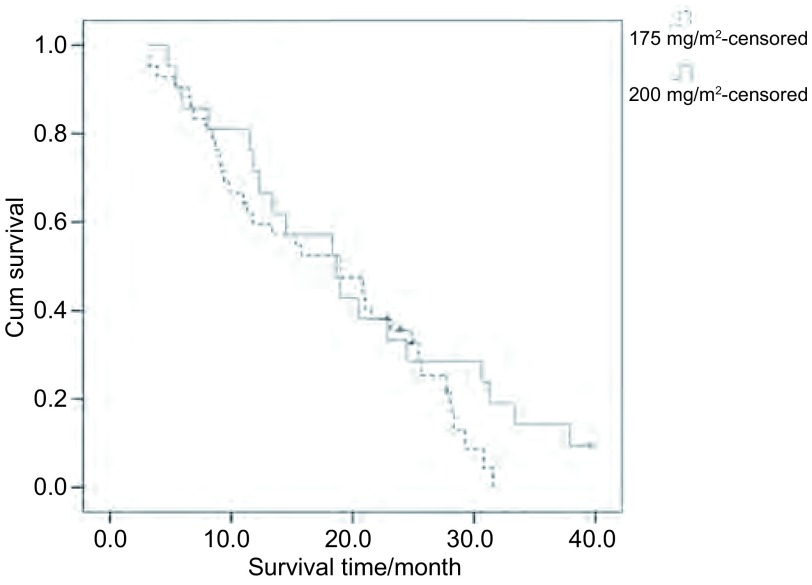
紫杉醇175 mg/m^2^及200 mg/m^2^联合卡铂化疗组患者生存曲线 Survival curves of paclitaxel (175 mg/m^2^) and paclitaxel (200 mg/ m^2^) groups

### 2.3 毒副反应

#### 2.3.1 血液系统毒性

紫杉醇200 mg/m^2^化疗组患者3/4级中性粒细胞减少的发生率高于175 mg/m^2^化疗组，分别为13例及14例（61.9% *vs* 33.3%, *P*=0.031）。200 mg/m^2^化疗组患者发生3/4级贫血共4例，175 mg/m^2^化疗组出现3/4级贫血为1例，200 mg/m^2^化疗组贫血发生率较175 mg/m^2^化疗组有增高趋势但无统计学差异（*P*=0.07）。3/4级血小板减少200 mg/m^2^化疗组为3例，175 mg/m^2^化疗组为1例，两组比较无统计学差异（*P*=0.201）。两组均未出现血液系统毒性相关性死亡（表 3）。

**3:** 紫杉醇175 mg/m^2^及200 mg/m^2^组患者毒副反应比较 Comparison of toxities between paclitaxel (175 mg/m^2^) and paclitaxel (200 mg/m^2^) groups

Toxity	Paclitaxel 175 mg/m^2^ (*n* =42)						Paclitaxel 200 mg/m^2^ (*n* =21)					*P*
	1	2	3	4	3+4 (%)		1	2	3	4	3+4 (%)	
Haematological toxicities												
Neutropenia	4	20	13	1	14（33.3%）		2	6	11	2	13 (61.9%)	0.031
Thrombocytopenia	9	7	1	0	1 (2.4%)		5	3	3	0	3 (14.3%)	0.201
Anemia	29	0	1	0	1 (2.4%)		10	1	4	0	4 (19.0%)	0.070
Nonhaematological toxicities												
Nausea and vomitting	6	1	1	0	1 (2.4%)		6	2	2	0	2 (9.5%)	0.530
Diarrhea	2	0	0	0	0		4	1	1	0	1 (4.8%)	0.333
Alopecia	20	5	1	0	1 (2.4%)		4	10	3	0	3 (14.3%)	0.201
Acral mumbness	10	6	0	0	0		12	3	1	0	1 (4.8%)	0.333
Arthragia	7	5	1	0	1 (2.4%)		8	6	2	0	2 (9.5%)	0.530
Fatigue	5	10	0	0	0		7	8	0	0	0	-
Skin rash	6	0	0	0	0		6	4	0	0	0	-
Constipation	5	4	0	0	0		8	3	0	0	0	-
Anorexia	6	1	0	2	2 (4.8%)		9	3	1	2	3 (14.3%)	0.426
Mouth ulcer	8	2	0	0	0		7	3	0	0	0	-
Venous embolism	0	0	0	1	1 (2.4%)		0	0	0	0	0	0.667
Edema of lower limbs	0	1	0	0	0		2	0	0	0	0	-
Heart	5	0	0	0	0		7	0	1	0	1 (4.8%)	0.333
Kidney dysfunction	0	0	0	0	0		0	0	0	0	0	-
Liver dysfunction	4	1	1	0	1 (2.4%)		2	2	0	0	0	0.667

#### 2.3.2 非血液系统毒性

毒副反应多为1/2级，两组3/4级恶心呕吐、肌肉酸痛、肢端麻木、腹泻、脱发、心脏毒性、纳差发生率均无统计学差异，但值得注意的是，上述3/4级毒性反应多发生于200 mg/m^2^化疗组。200 mg/m^2^化疗组1例患者出现3级室性早搏。175 mg/m^2^化疗组1例患者出现3级肝功能异常，该患者既往有慢性乙型肝炎病史，1例患者发生深静脉血栓。两组均未出现3/4级便秘、肾功能异常、口腔溃疡、下肢水肿、皮疹及乏力（表 3）。

### 2.4 后续治疗

多数患者疾病进展后均接受后续治疗，紫杉醇175 mg/m^2^及200 mg/m^2^化疗组分别有23例（54.8 %）及12例（57.1 %）患者进展后接受化疗，治疗情况见表 1。紫杉醇175 mg/m^2^化疗组有24例患者接受二线或二线后EGFR-TKI靶向治疗，200 mg/m^2^化疗组有11例接受表皮生长因子受体酪氨酸激酶抑制剂（epidermal growth factor receptor-tyrosine kinase inhibitors, EGFR-TKI）治疗（57.1% *vs* 52.4%, *P*=0.72）。

## 3 讨论

紫杉醇的抗肿瘤作用机理为促进微管聚合和稳定已聚合的微管结构，导致细胞周期停滞于G2/M期，使快速分裂的肿瘤细胞出现生长抑制而死亡，另外也具有促进肿瘤细胞凋亡及免疫调节功能。根据以往的临床试验研究，紫杉醇联合卡铂化疗一线治疗晚期NSCLC，客观有效率为27%-40%，MST为10个月-12个月，1年生存率为30%-54%^[2-5]^。在一项比较欧洲患者使用不同剂量紫杉醇联合卡铂方案治疗晚期NSCLC的多中心临床研究中，紫杉醇175 mg/m^2^及225 mg/m^2^剂量组的客观有效率分别为25.6%与31.8%（*P*=0.733），TTP分别为4.3个月与6.4个月（*P*=0.044），1年生存率分别为37%与44%（*P*=0.35），MST分别为9.5个月与11.4个月（*P*=0.16），225 mg/m^2^组发生3/4级中性粒细胞下降的比率较175 mg/m^2^组增高，分别为12.1%与5%（*P*=0.038）。研究^[6]^提示较高剂量的紫杉醇联合卡铂化疗可延缓欧洲患者疾病进展时间，但血液系统毒副反应发生率明显增加，ORR、1年生存率及MST两组比较则无统计学差异。

药物临床试验是指导晚期NSCLC患者治疗的主要依据，目前大多数循证医学的依据来自于欧美国家的资料。在多数情况下这些临床循证医学的证据对亚洲包括中国人种的肺癌治疗具有较好的借鉴作用，但由于试验设计、入选标准、受试者一般情况、申办方监查频率、人群相关药代动力学、药效学、药物遗传学等可能存在差异，针对单一人群的临床试验结果可能并不完全适用于另一人群。

Sekine等^[7]^回顾性分析了入选标准相同的不同地区含铂方案治疗晚期NSCLC的Ⅲ期临床试验，这些临床试验的诊断分期、疗效、毒副反应评价方法均一致，在接受紫杉醇联合卡铂方案化疗的分组中，日本和欧洲患者紫杉醇剂量为200 mg/m^2^，美国患者为225 mg/m^2^，卡铂剂量均为AUC 6。结果显示88%的日本患者出现3/4级中性粒细胞下降，而欧洲及美国患者出现3/4级中性粒细胞下降的比例分别为15%-51%和6%-65%；血小板下降比例日本患者为11%，欧美患者为2%-10%，非血液系统毒性发生率无明显差异。日本患者ORR为32%，1年生存率为51%，MST为12.3个月，欧美患者分别为17%-46%、32%-43%及7.8个月-11.0个月。Gandara等^[8]^前瞻性研究日本（LC00-03）及美国（S0003）含紫杉醇卡铂方案治疗组的Ⅲ期临床试验，LC00-03及S0003组患者在相同化疗剂量下，化疗后出现3/4级中性粒细胞减少的比例为70%及38%（*P* < 0.001），3/4级血小板下降比例为7%及6.5%（*P*=0.31），非血液系统毒性发生率两组无统计学差异。ORR分别为37%及33%，TTP为6个月及4个月（*P*=0.04），MST为14个月及9个月（*P*=0.000, 6），1年生存率为57%及37%（*P*=0.000, 4）。

以上研究显示日本患者接受紫杉醇联合卡铂方案化疗生存获益优于欧美患者，但相关血液系统毒性发生率明显增高。出于对患者安全性与耐受性的考虑，国内临床医师多选择偏低于标准治疗剂量的方案。采用不同剂量紫杉醇联合卡铂方案化疗对国内晚期NSCLC患者毒副反应、治疗反应及生存预后影响的报道目前较少。本组63例病例，分别使用紫杉醇200 mg/m^2^及175 mg/m^2^联合卡铂方案化疗，两组未出现毒副反应相关性死亡。与国外相关报道类似，紫杉醇200 mg/m^2^组3/4级中性粒细胞下降发生率高于175 mg/m^2^组，分别为61.9%与33.3%（*P*=0.031）。3/4级贫血及血小板下降发生率200 mg/m^2^组均高于175 mg/m^2^组，但无统计学差异。两组非血液系统毒性多为可耐受的1/2级，3/4级毒副反应发生率无明显差异，但较多发生于200 mg/m^2^化疗组。紫杉醇200 mg/m^2^化疗组客观有效率为33.33%，175 mg/m^2^化疗组为28.57%，与以往文献报道ORR范围一致，200 mg/m^2^化疗组ORR较高，但无统计学差异。紫杉醇175 mg/m^2^化疗组中位TTP为6.7个月，200 mg/m^2^化疗组中位TTP为7个月。175 mg/m^2^化疗组MST为18.7个月，200 mg/m^2^化疗组为19个月。1年生存率、2年生存率两组分别为61.9%、31.0%和66.7%、33.3%，生存预后数据两组均无统计学差异。

抗肿瘤药物被认为对种族因素具有较高敏感性，这是由于此类药物药效与安全性曲线较陡、治疗剂量范围狭窄及非线性的药代动力学，特别是由于编码药物代谢酶的基因遗传多态性，导致抗肿瘤药物的代谢及活性在不同种族间存在差异^[9]^。已知UDP-葡萄糖醛基转移酶（UDP Galactosyltransferase, UGT）1A1^*^28（一种*UGT1A1*基因启动子区域多态性）纯合型患者接受伊立替康（irinotecan）化疗后4级中性粒细胞下降的发生率为40%-57%，而野生型基因患者的发生率低于15%，且亚洲患者UGT1A1^*^28纯合型比例低于欧美患者^[10, 11]^。紫杉醇在体内代谢消除主要由多种细胞色素P450（cytochrome P450）亚型如CYP2C8、CYP3A4及CYP3A5介导。Gandara等^[8]^比较日本及欧美患者与紫杉醇药物代谢酶及DNA损伤修复相关基因多态性，发现CYP3A4^*^1B、CYP3A5^*^3C、ERCC1 118（excision repair cross-complementation group 1，核苷酸切除修复交叉互补组1）、*ERCC2* *K751Q*及*CYP2C8 R139K*基因分布在日本及欧美人群中存在差异，CYP3A4^*^1B多态性与无疾病进展生存期（progress free survival, PFS）相关（HR=0.36, 95%CI: 0.14-0.94, *P*=0.04）。ERCC2 K751Q与反应率相关（HR=0.33, 95% CI: 0.13-0.83, *P*=0.02）。4级中性粒细胞下降在ABCB1（即MDR-1, multi-drug resistance gene，多药耐药基因）3425C→ T基因变异中具有一定趋势，但无统计学意义（HR=1.84, 95%CI: 0.77-0.83, *P*=0.19），研究未发现与患者生存预后相关的基因多态性，可能需进一步扩大受试人群样本后分析。以上药物遗传学研究提示在某一种族人群中获得某种抗肿瘤药物的药理性质及推荐剂量后相关试验结果是否适用于另一种族，需要进行Ⅰ期、必要时Ⅱ期临床试验，以便对该药物的药效学、药代动力学及药物遗传学等方面进行深入研究。而Ⅰ期及Ⅱ期临床试验结果有助于确定大样本多中心临床研究的试验及用药方案，对深入探索新型抗肿瘤药物的毒性及疗效颇有裨益。

本研究两种剂量化疗组ORR与以往文献报道类似，TTP略高于LC00-03试验日本患者TTP。两组生存率、MST均高于以往文献报道，可能与研究病例数较少、入选患者ECOG评分良好（0分-1分）、受试者来自单一临床研究单位，具有一定选择性有关。两组患者多接受二线及二线后治疗，值得注意的是，近年来靶向治疗已全面融入晚期NSCLC患者的治疗，EGFR-TKI治疗晚期NSCLC的优势人群为腺癌、女性、不吸烟及亚裔患者。EGFR突变阳性的NSCLC患者对EGFR-TKI治疗的反应率、TTP及生存时间优于突变阴性患者。中国人EGFR突变率约为30.04%，其中腺癌患者突变率为44.1%，非腺癌患者突变率为9.2%，女性患者突变率为42.9%，男性患者为23.1%^[12]^。本组紫杉醇175 mg/m^2^及200 mg/m^2^化疗组腺癌比例均较高分别为78.6%与81.0%，且较多患者在疾病进展后接受二线或三线EGFR-TKI治疗（57.1% *vs* 52.4%），这也可能是生存率、MST高于以往文献报道的原因。

综上所述，较低剂量紫杉醇（175 mg/m^2^）联合卡铂一线治疗晚期NSCLC患者可减少中性粒细胞下降毒副反应发生率，且患者疗效及生存时间并不劣于较高剂量化疗组。合理的二线及二线后治疗策略、联合分子靶向治疗，可改善晚期NSCLC患者预后。临床医师在选择肺癌患者治疗方案时，应结合相关临床及基础试验数据资料，注意抗肿瘤药物治疗可能存在的种族差异。进行规范的Ⅰ/Ⅱ期临床试验，探索中国患者对相关抗肿瘤药物的药效学、药代动力学及药物遗传学特点，是确定合理用药方案与剂量的最优途径。
